# Chirality and pH Influence the Self-Assembly of Antimicrobial Lipopeptides with Diverse Nanostructures

**DOI:** 10.1021/acsabm.4c00664

**Published:** 2024-07-23

**Authors:** Anindyasundar Adak, Valeria Castelletto, Bruno Mendes, Glyn Barrett, Jani Seitsonen, Ian W. Hamley

**Affiliations:** † School of Chemistry, Pharmacy and Food Biosciences, University of Reading, Whiteknights, Reading RG6 6AD, U.K.; ‡ School of Biological Sciences, University of Reading, Whiteknights, Reading RG6 6AH, U.K.; § Nanomicroscopy Center, Aalto University, Puumiehenkuja 2, FIN-02150 Espoo, Finland

**Keywords:** lipopeptides, peptide amphiphiles, chirality, pH sensitivity, micelles, nanofibers, nanotubes, antimicrobial peptides

## Abstract

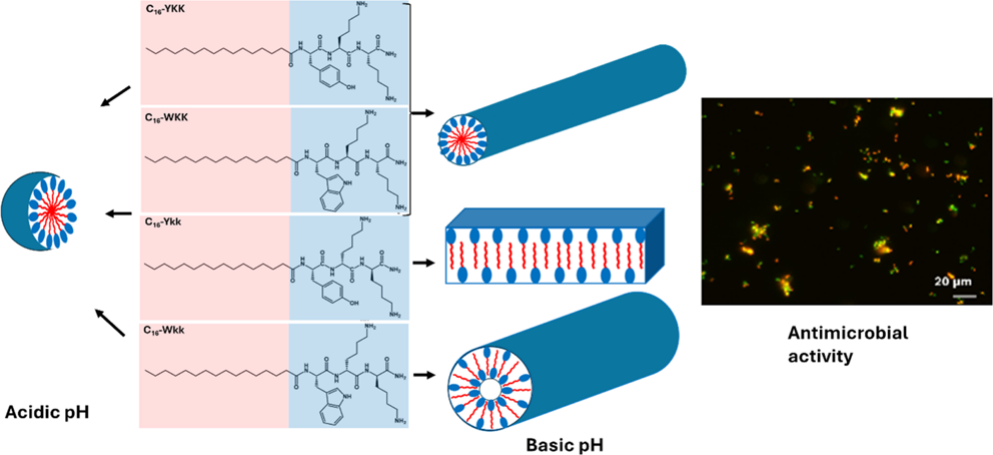

Chirality plays a crucial role in the self-assembly of biomolecules in nature. Peptides show chirality-dependent conformation and self-assembly. Lipidation of peptides occurs in vivo and has recently been exploited in designed conjugates to drive self-assembly and enhance bioactivity. Here, a library of pH-responsive homochiral and heterochiral lipidated tripeptides has been designed. The designed lipopeptides comprise homochiral C_16_–YKK or C_16_–WKK (where all the amino acids are l-isomers), and two heterochiral conjugates C_16_–Ykk and C_16_–Wkk (where the two lysines are d-isomers). The self-assembly of all the synthesized lipopeptides in aqueous solution was examined using a combination of spectroscopic methods along with cryogenic-transmission electron microscopy (cryo-TEM) and small-angle X-ray scattering (SAXS). Interestingly, it was observed that at acidic pH all the lipopeptides self-assemble into micelles, whereas at basic pH the homochiral lipopeptides self-assemble into nanofibers, whereas the heterochiral lipopeptides self-assemble into nanotapes and nanotubes. A pH switch was demonstrated using a thioflavin T fluorescence probe of β-sheet structure present in the extended structures at pH 8. We demonstrate that both chirality and pH in lipopeptides influence the self-assembly behavior of the model tripeptides, which also show promising bioactivity. Good cytocompatibility is observed in hemolytic assays and antimicrobial activity against both Gram-negative and Gram-positive bacteria is shown through the determination of minimum inhibition concentration (MIC) and minimum bactericidal concentration (MBC) values and live/dead bacteria staining assay.

## Introduction

The self-assembly of chiral molecules plays a critical role in the origin of life and its subsequent evolutionary processes on Earth.^1,2^ To understand the beginning of life and its evolution, the self-assembly of chiral building blocks must be considered.^3,4^ A range of noncovalent interactions aid the organization of chiral building blocks into defined conformations and structures to facilitate the functioning of complex biological systems such as proteins, single-stranded helices of RNA, double-stranded helices of DNA, phospholipid bilayers of lipid cell membranes, triple strands of collagen, etc., which are the essential components of living matter. Non-covalent interactions such as hydrogen bonding, ionic interactions, π–π stacking, and van der Waals forces, play a significant role in the self-assembly processes of these complex biological systems.^5,6^

Mimicking this complex self-assembly process within biological systems is very challenging and it is necessary to examine the role of the diverse noncovalent interactions leading to the self-assembly of the chiral building blocks. Nature generally prefers homochiral assembly in a sustainable self-replication cycle to create complex biological systems.^7,8^ Synthetic peptide chemistry opens new routes to self-assembly pathways, to mimic or go beyond natural self-assembly phenomena. This could lead to a better understanding of complex biological systems. Proteins and peptides in most living organisms are mainly based on l-amino acids, and homochirality is important in the self-assembly process (protein folding, assembly of complexes, aggregation, etc.). Peptides are versatile biocompatible building blocks, and a better understanding of the influence of chirality on their functionality is required.^9^ For example, Wang et al. designed three pairs of peptides with varying molecular chirality and studied the self-assembly pattern of their nanostructures and the handedness of the supramolecular structures (twisted fibrils).^10^ Clover et al. showed that changing the chirality of amino acids in the designed peptide sequences leads to the formation of various nanostructures.^11^ In another example, tripeptides were designed with variation of amino acid chirality to mimic supramolecular assembly.^8^ Kurbasic et al. designed heterochiral tetrapeptides containing amyloidogenic Phe-Phe sequences, which self-assemble to form hydrogels for hydrolase mimicry.^12^

Chiral lipopeptides (peptide amphiphiles, PAs) which consist of hydrophobic and hydrophilic regions, are known to form various nanostructures due to noncovalent interactions.^13^ Generally during the self-assembly process, the hydrophobic part aggregates in the core, while the hydrophilic part forms the outer layer. Depending on the peptide sequence, length of hydrophobic lipid chain, and overall amphiphilic character, lipopeptides exhibit various types of self-assembled nanostructures such as micelles,^14^ vesicles,^15^ nanofibrils,^16^ nanotubes,^17^ nanosheets,^18^ nanoribbons,^19^ etc. Micelle formation in PAs results from the hydrophobic effect which leads to the sequestration of the lipid chains inside the core of the self-assembled structure.^20^ Nanofibril formation of PAs is driven by the β-sheet secondary structure of the peptide moiety, although the ordering of the lipid chains is also important.^13,21^ In nanotubes formed by PAs, the hydrophobicity of the lipid chain, and the antiparallel β-sheet secondary structure of the peptide moiety (adopted in most lipopeptide nanotubes) are important in the self-assembly process for the case of nanotubes formed by wrapped nanosheets.^22,23^ Similar interactions are responsible for the self-assembly of PAs into nanosheets (which can be considered to be a type of unwrapped nanotubes).^18,24^ Changing the peptide sequence from homochiral to heterochiral in lipopeptides can influence the handedness of the supramolecular structure. For example, Xie et al. showed that by changing the chirality of the peptide sequences in lipopeptides, the helicity of self-assembled nanofibers can be reversed.^25^

Antimicrobial peptides (AMPs) have great potential to combat bacterial pathogens, especially in the age of multidrug resistance. The mechanism of antimicrobial activity arises from the bacterial membrane permeabilization and disruption by the AMP.^26,27^ One category of AMPs can be found within the innate immune systems of both animal and plant kingdoms, where they play a crucial role in host defense mechanisms.^28^ Another type of AMP comprises designed or bioinspired molecules where the hydrophobic part of the AMP binds with the hydrophobic region of the lipid membrane, and positively charged residues interact with the negatively charged bacterial membrane, which ultimately leads to bactericidal activity.^29−31^ Lipopeptides also exhibit antimicrobial activity since their amphiphilic character can perturb the bacterial membrane, resulting in bacterial death.^32,33^ Along with the antimicrobial activity of lipopeptides, they also self-assemble into various nanostructures,^34^ for example, di-lipidated KKK or KEK peptides form ribbon-like structures,^35^ while C_16_–KKFF and C_16_–KKF form spherical micelles.^36^ Lysine-rich lipopeptides consisting of amyloid peptide fragments were shown to exhibit promising antimicrobial and wound-healing properties.^37^ Makovitzki et al. found significant antimicrobial activity in cationic lipopeptides with homochiral or heterochiral sequences, although the heterochiral lipopeptides did not exhibit any significant antimicrobial activity.^38^ Sikorska et al. studied the self-assembly and binding interaction between lipopeptides bearing short lysine-rich sequences and membranes, and they observed that both electrostatic and hydrophobic interactions played a crucial role in the interaction of lipopeptides with the lipid bilayer.^39^ The cellular uptake of two cell-penetrating peptides was compared, highlighting the role of the C-terminal tripeptide WKK.^40^ Laverty and co-workers designed cationic lipopeptides, consisting of tryptophan and lysine residues which displayed significant antimicrobial activity against multidrug-resistant pathogenic bacteria and fungi.^32^ Gong et al. developed a lipopeptide-hydrogel based on PA molecules containing a C_12_ lipid chain and a (IIKK)_2_ peptide, which exhibited significant activity in treating *Helicobacter pylori* infection.^41^ Tan et al. developed a lipopeptide hydrogel consisting of a C_16_–WIIIKKK peptide, which effectively inhibited bacterial growth by releasing the WIIIKKK antimicrobial peptide.^42^

Inspired by the above results, we designed antimicrobial lipopeptides containing tripeptide sequences comprised of cationic and aromatic amino acid residues, where we have also altered the chirality of the lysine residues to build both homochiral and heterochiral lipopeptides and explored their self-assembly as well as antimicrobial activity. In our recently published report, we described the synthesis of the four lipopeptides C_16_–YKK, C_16_–WKK, C_16_–Ykk, and C_16_–Wkk (here, C_16_ represents a palmitoyl lipid chain and Y, W, K, and k stand for tyrosine, tryptophan, lysine, and d-lysine respectively).^43^ We also reported on the self-assembly of spherical micelles of all four lipopeptides in aqueous solutions at native pH 4.6, based on cryo-TEM imaging and small-angle X-ray scattering (SAXS). Furthermore, from spectroscopic studies such as circular dichroism (CD) and Fourier-transform infrared spectroscopy (FTIR), it was observed that the hydrophilic peptide moieties in the coronas adopt an unordered conformation. All the lipopeptides exhibited good cytocompatibility with fibroblasts at low lipopeptide concentrations, whereas at the highest concentrations of lipopeptide (above the critical aggregation concentration, CAC, of the lipopeptides) cytotoxicity was observed. They exhibited significant antimicrobial activity against both Gram-negative and Gram-positive bacteria in preliminary antimicrobial assays.^43^

Here, we report on the effect of chirality in the lipopeptides and variation of solution pH, and how these can be used to tune the peptide assembly. Extended nanostructures including fibrils, nanotapes, and nanotubes are observed at pH 8 in contrast to the micelles formed at native pH 4.6. The formation of distinct structures (fibrils vs nanotapes/nanotubes) is ascribed to differences in molecular packing arising in homochiral compared to heterochiral molecules, as probed by NMR and other spectroscopic methods. Along with a comprehensive study of peptide conformation and self-assembly we also further examine antimicrobial activity and hemocompatibility of the lipopeptides. The two heterochiral lipopeptides containing d-lysine can be expected to have greater stability in vivo (due to reduced proteolysis of these non-native residues) and hence are particularly promising antimicrobial activities, here this is compared with that of the l-lysine homologues.

## Experimental Section

### Materials

Rink amide resin, trifluoroacetic acid (TFA), and *N*,*N*′-dimethylformamide (DMF), diethyl ether, methanol, piperidine, HPLC grade water, HPLC grade acetonitrile were purchased from Thermo-Fisher. The Fmoc amino acids, triisopropylsilane (TIS), diisopropylethylamine (DIPEA), and *O*-(1-benzotriazolyl)-1,1,3,3-tetramethyluronium hexafluorophosphate (HBTU), were purchased from Sigma-Aldrich.

### Synthesis of Lipopeptides

The details of the synthesis of the four lipopeptides and the associated characterization data are provided in our recent report.^43^

### Sample Preparation

A measured amount of lipopeptide was dissolved in water to make a sample with a defined concentration in wt %, and pH was measured using a Mettler Toledo FiveEasy pH meter with a Sigma-Aldrich micro-pH combination electrode (glass body), and the pH was found to be approximately 4.6 as native pH for all four lipopeptides. For basic pH 8 solutions, a few drops of 1 wt % NaOH solution were added, confirmed using the pH meter.

### CD Spectroscopy

The circular dichroism (CD) spectra of the lipopeptides were measured using a Chirascan spectropolarimeter (Applied Photophysics, Leatherhead, U.K.) connected to a thermal controller. For each scan of the samples, 0.1 mm quartz cells were used. Each scan was recorded three times from 180 to 280 nm, with 0.5 nm step, 1 nm bandwidth, and 1 s collection time per step. Water was used as the control for background subtraction.

### Fourier Transform Infrared (FTIR) Spectroscopy

For recoding the FTIR spectra, a Thermo-Scientific Nicolet iS5 instrument was used, which has a DTGS detector, and a Specac Pearl liquid cell containing CaF_2_ plates to house the sample solution. For each spectrum, a total of 128 scans for each sample were recorded over the range of 900–4000 cm^–1^.

### Fluorescence Spectroscopy

For the fluorescence experiments, a Varian Cary Eclipse spectrofluorometer was used, and the samples were placed in 4 mm inner-width quartz cuvettes. For experimental settings, excitation and emission bandwidths of 2.5 nm were used. All the experiments were carried out at 25 °C. The CAC value for all the samples was determined by fluorescence experiments with Thioflavin T (ThT). ThT is well known to bind to amyloid fibrils, and can be used to determine CAC values.^44−46^ First a stock solution (5 × 10^–4^ wt %) ThT solution was prepared and using this solution, various concentrations of samples were prepared. The scan of the fluorescence spectra (λ_ex_ = 440 nm) was taken from 460 to 670 nm.

The CAC value of the samples was calculated by plotting *I*/*I*_0_ versus concentration (in wt %). Here *I*_0_ is the maximum intensity for the control, i.e., ThT solution without the sample, and *I* signifies the maximum fluorescence intensity of ThT at a given concentration of lipopeptide.

### Cryogenic-Transmission Electron Microscopy (Cryo-TEM)

The Cryo-TEM imaging was performed using a field emission cryo-electron microscope (JEOL JEM-3200FSC), operating at 200 kV. To take the TEM images bright field mode with a zero-loss energy filter (omega type) was used, with a slit width of 20 eV. A Gatan UltraScan 4000 CCD camera was used to record the micrographs. During the entire experiment, the sample was kept at −187 °C. An automated FEI Vitrobot device was used to blot and vitrify samples on Quantifoil 3.5:1 holey carbon copper grids with a hole size of 3.5 μm. Before taking the images, grids were first plasma cleaned using a Gatan Solarus 9500 plasma cleaner, followed by transferring to the environmental chamber of a FEI Vitrobot at room temperature and 100% humidity. For sample spotting on the grid, 3 μL of sample solution was taken and spread on the grid. It was blotted twice and then vitrified in a 1:1 mixture of liquid ethane and propane at a temperature of −180 °C. The grids with vitrified sample solution were maintained at liquid nitrogen temperature and then cryo-transferred to the microscope.

### Small-Angle X-ray Scattering (SAXS)

Experiments were carried out on beamline B21 at Diamond (Didcot, U.K.). The sample solutions were placed into the 96-well plate of an EMBL BioSAXS robot and then injected via an automated sample exchanger into a quartz capillary (1.8 mm internal diameter) in the X-ray beam. To avoid air scattering, the quartz capillary was kept inside a vacuum chamber. After the sample was injected into the capillary and reached the X-ray beam, the flow was stopped during the SAXS data acquisition. Beamline B21 operates with a fixed camera length (3.9 m) and fixed energy (12.4 keV). A PILATUS 2M detector was used to record SAXS patterns, and data was processed using dedicated beamline software ScÅtter.

### 2D NOESY NMR study

For the two-dimensional NMR (2D NMR) study of lipopeptides, 1 wt % solutions of lipopeptides in D_2_O were prepared at pH 8 (by adding a few drops of 1 wt % NaOD solution). Next, a standard 256 NOESY scans were recorded for each lipopeptide solution using a 400 MHz Bruker Nanobay spectrometer. The graphs were plotted and analyzed in Mnova software.

### Minimum Bactericidal Concentration (MBC)

Two Gram-negative strains (*Escherichia coli* K12 and *Salmonella enterica* NCTC 5188) and one Gram-positive strain (*Staphylococcus aureus* ATCC 12600) were employed to evaluate the potential bactericidal activity of tested lipopeptides. A single colony from each strain, pre-streaked onto an LB (lysogeny broth) agar plate, was inoculated into 5 mL of LB broth and incubated at 37 °C with shaking (250 rpm) for 18 h. Then, the bacterial culture was adjusted to 5 × 10^6^ CFU mL^–1^ mid-log phase in Mueller-Hinton broth (MHB), and aliquots were transferred to individual wells of a U-bottom 96-well microplate with increasing concentrations of lipopeptides (0–1000 μg mL^–1^). Included positive and negative controls consisted of 0.97 μg mL^–1^ ciprofloxacin or 1× PBS in MHB, respectively. After 18 h of incubation at 37 °C, 10 μL from each well was spot-inoculated onto LB agar and plates were further incubated at 37 °C overnight to assess the MBC which refers to the lowest concentration of an antibacterial agent required to kill bacteria (here denoted by colony growth on the plate).

### Assessment of Peptide-mediated Hemolysis

The in vitro toxicity of the lipopeptides to human red blood cells (hRBCs) was conducted by following, with modifications, an absorbance-based assay described by Oddo et al.^47^ In brief, one volume of hRBCs was diluted in three volumes of 1× sterile phosphate-buffered saline (PBS) and centrifuged at 700*g* for 10 min. The supernatant was carefully discarded using a Pasteur pipette to avoid disturbing the pellet which was then resuspended in three volumes of PBS. This wash step was repeated at least three times. Following this, a stock solution containing 0.5% hRBCs (v/v) was dispensed into individual wells of a 96-well microtiter plate containing PBS and lipopeptides at concentrations ranging from 15.62 to 1000 μg mL^–1^. PBS and 1% membranolytic detergent (Triton X-100), to replace peptides, were employed as negative and positive controls, respectively. Microtiter plates were then incubated at 37 °C for 1 h and spun in a microplate centrifuge at 1000*g* for 10 min to pellet hRBCs. The resulting supernatant was collected and transferred to a clean 96-well flat-bottomed microplate, and the absorbance (optical density, OD) of each well was measured at 414 nm using a Tecan Spark multimode plate reader. The rate of hemolysis was calculated by applying the formula: Hemolytic activity (%) = 100(OD_lipopeptide_ – OD_PBS_)/ (OD_Triton_ – OD_PBS_). The dose-response curve was obtained using GraphPad Prism (v.8.0.2) software. Additionally, the selectivity index (SI) values were determined as HC_50_/MIC where HC_50_ indicates the concentration for 50% hemolytic activity and MIC indicates minimum inhibitory concentration for bacteria (reported previously).^43^ All experiments were performed in triplicate from three independent experiments.

### Assessment of the Impact of Lipopeptides on Membrane Integrity

A bacteria Live/Dead staining kit (PromoKine) was employed to gain insight into the possible membrane disruption activity of tested lipopeptides. In summary, we used two color fluorescence dyes with varying ability to cross the cytoplasmatic membranes of bacteria. DMAO is a green-fluorescent nucleic acid dye which stains the cell membranes of both live and dead bacteria, while the red fluorescing EthD-III dye selectively stains dead bacteria with compromised membranes. With appropriately calculated volumes of dyes, live viable bacterial cells fluoresce green whilst dead compromised cells fluoresce red. For this assessment, a total of 5 × 10^6^ mL^–1^ exponential phase bacterial cells were incubated with two-fold MIC of lipopeptides for 1 h at 37 °C. Negative and positive controls consisted respectively of the addition of PBS and Triton-X100 (1%) to replace lipopeptides. Afterwards, the sample was centrifuged at 1000*g* for 10 min. The pellet was resuspended in 100 μL of 0.85% buffered NaCl with the epifluorescence dyes, following the kit instructions, and incubated for 15 min at room temperature. The samples were examined under a Nikon Eclipse Ti2 inverted microscope.

## Results and Discussion

**Figure 1:**
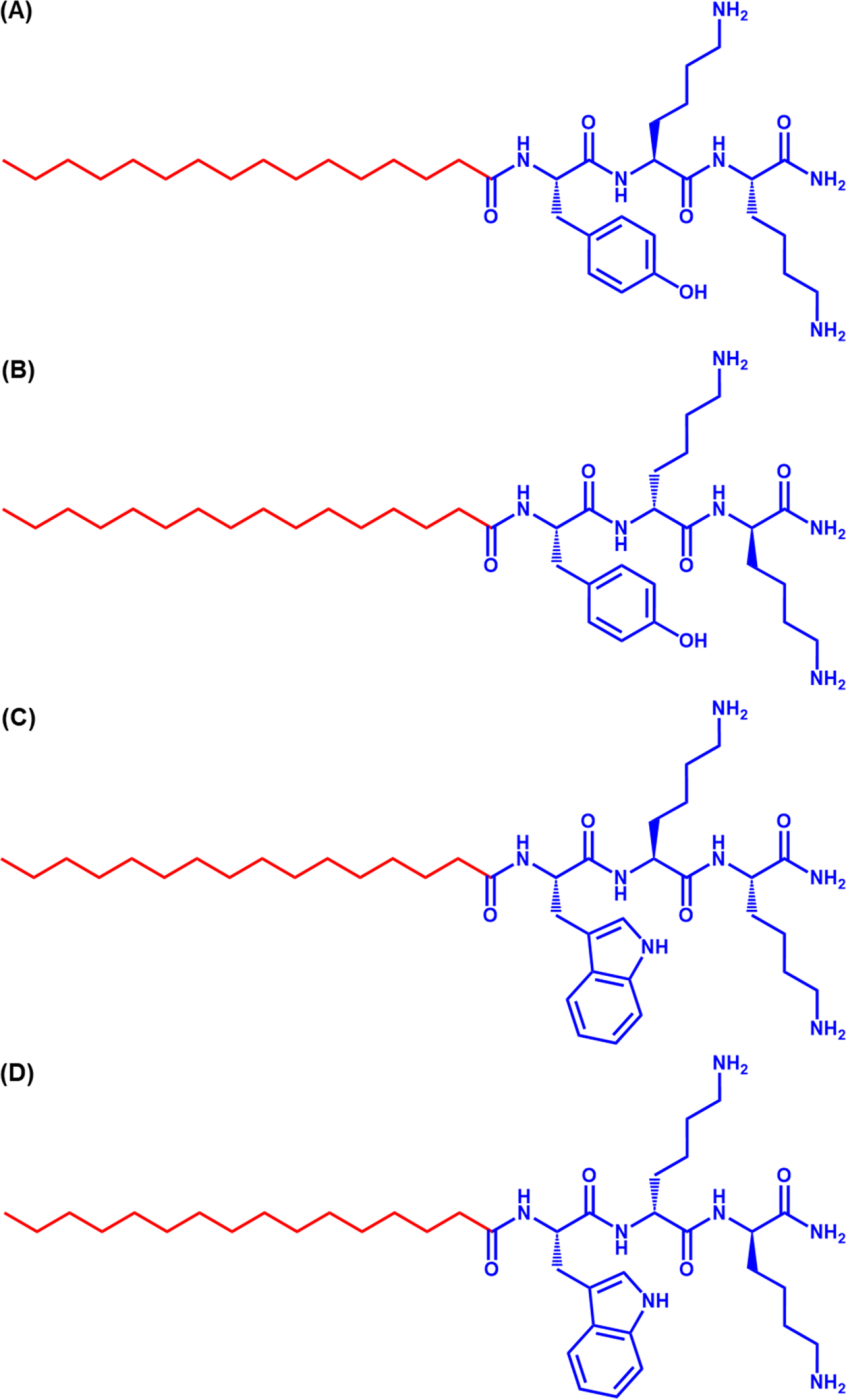
Structure of lipopeptides (A) **P1**, (B) **P1D**, (C) **P2**, (D) **P2D**. The C terminal was converted to amide for all the lipopeptides. Here, the red and blue parts correspond to lipid and tripeptide, respectively.

We studied the self-assembly and bioactivity (hemocompatibility and antimicrobial activity) of the four lipopeptides C_16_–YKK, C_16_–Ykk (k: d-lysine), C_16_–WKK and C_16_–Wkk with structures shown in Figure 1. Here, they are abbreviated as **P1, P1D, P2, P2D**, respectively. These peptides were synthesized in our laboratory based on the standard Fmoc solid-phase synthesis strategy. After purification, the collected products were analyzed using reversed-phase high-performance liquid chromatography (RP-HPLC) and matrix-assisted laser desorption ionization-time of flight mass spectrometry (MALDI-TOF MS). The results indicated the correct mass and high purities (more than 98%).^43^

We have already explored the self-assembly characteristic of the lipopeptides **P1, P1D, P2, and P2D** in water at pH 4.6 in our recent publication,^43^ and showed that micelles form at pH 4.6, as evidenced by cryo-TEM imaging and SAXS. Inspired by the above results, we were motivated to explore the self-assembly characteristics of these lipopeptides at higher pH i.e., basic pH 8, and to examine the mechanism of self-assembly of these lipopeptides.

**Figure 2:**
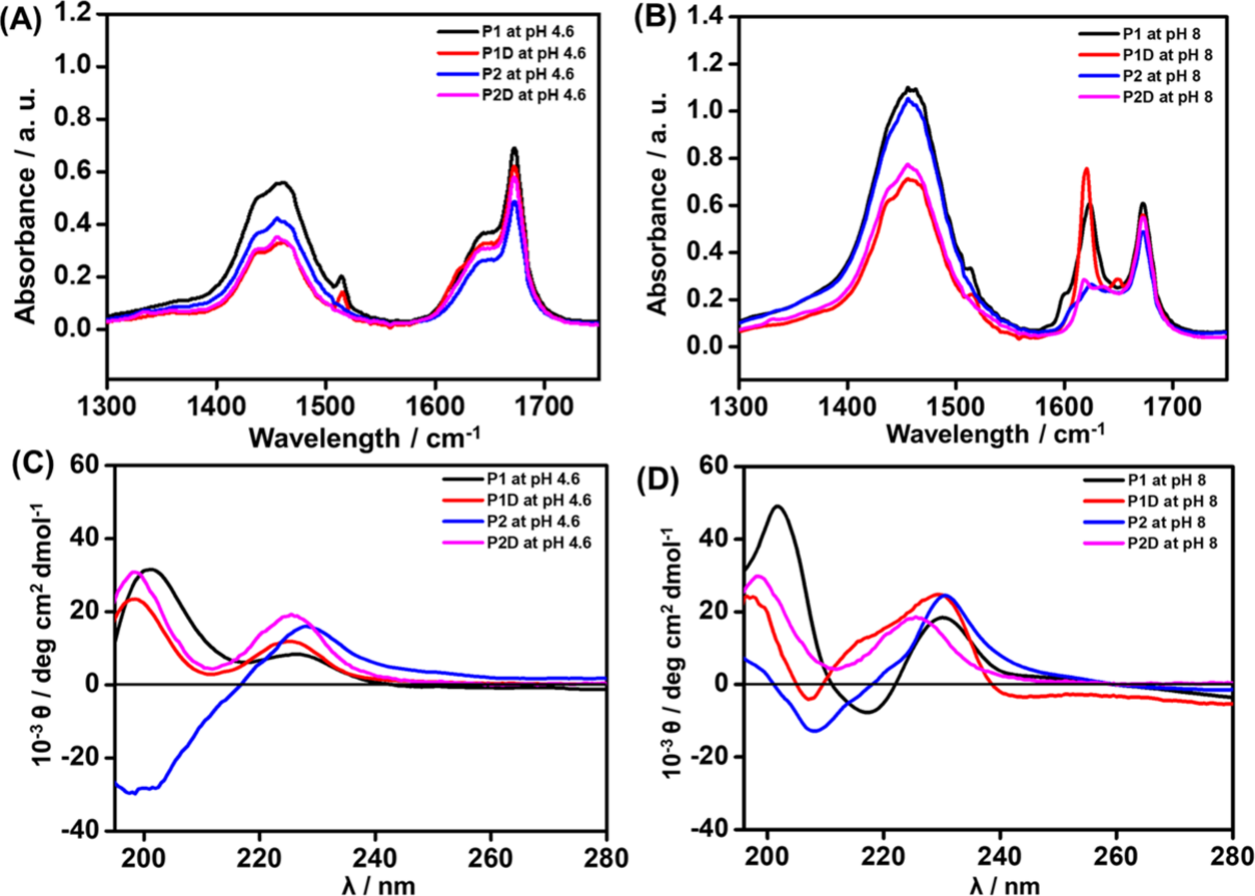
FTIR spectra of 1 wt % aqueous solution of lipopeptides (A) at pH 4.6, (B) at pH 8. CD spectra of 1 wt % aqueous solution of lipopeptides (C) at pH 4.6, (D) at pH 8.

The conformation of the peptides at pH 8 was first examined using spectroscopic methods. FTIR spectra for these lipopeptides at pH 8, are shown in Figure 2A,B where the amide-I′ and -II′ regions are shown, the amide-I′ region being important to characterize the secondary structure of peptides. From the results, bands in the range of 1320–1740 cm^–1^ were observed for **P1, P1D, P2, and P2D**. The strong peak at 1620 cm^–1^ (Figure 2B) suggests the formation of a β-sheet secondary structure for the lipopeptides.^48−50^ This peak is absent in the case of 1 wt % aqueous solutions of these lipopeptides at pH 4.6 (Figure 2A) (when the peptides have a disordered/random coil structure^43,48,49^), which signifies that changing the pH from acidic to basic triggers the β-sheet secondary structure of these lipopeptides, which can ultimately lead to a transition to a different nanostructure at basic pH (as discussed below) compared to previously reported micelles at lower pH 4.6.^43^ Since all the lipopeptides contain an aromatic side chain, a broad peak was observed in the amide II′ region centered around 1400–1500 cm^–1^ (Figure 2A,B).^49^ From Figure S1A,B, the peaks corresponding to the CH/CH_2_/CH_3_ stretching modes of the lipid chains in lipopeptides were observed (at ∼2850 and ∼2925 cm^–1^), which confirms the presence of the lipid chain in the lipopeptides. These are enhanced at pH 8, due to the higher degree of ordering of the lipid chains in the extended β-sheet nanostructures.

CD spectroscopy was also employed to probe the secondary structure. The spectra for 1 wt % aqueous solutions of the lipopeptides at pH 4.6, suggest an unordered coil conformation of lipopeptides (Figure 2C). Interestingly the spectra for homochiral lipopeptide **P1** and **P2** solutions at pH 8, displayed a minimum with a negative band at 218 nm and positive bands near 200 nm, which suggests a β-sheet secondary structure (Figure 2D). The spectra for heterochiral molecules **P1D** and **P2D** at pH 8 show a positive minimum at 210–218 nm and a maximum near 200 nm consistent with β-sheet secondary structure with inverted handedness (Figure 2D). The distinct features of the CD spectra of **P2** and **P2D** arise from the tryptophan residue which plays a crucial role in the self-assembly process of lipopeptides with a greater degree of peptide backbone ordering.^51^ The CD spectra show that in 1 wt % aqueous solution **P1, P1D**, **P2**, and **P2D** at pH 8 adopt a β-sheet secondary structure (Figure 2D).^52^ All the CD spectra exhibited a band in the range of 225–230 nm, which appeared because of the presence of tyrosine for **P1** and **P1D** and tryptophan for **P2** and **P2D**.^53^

**Figure 3:**
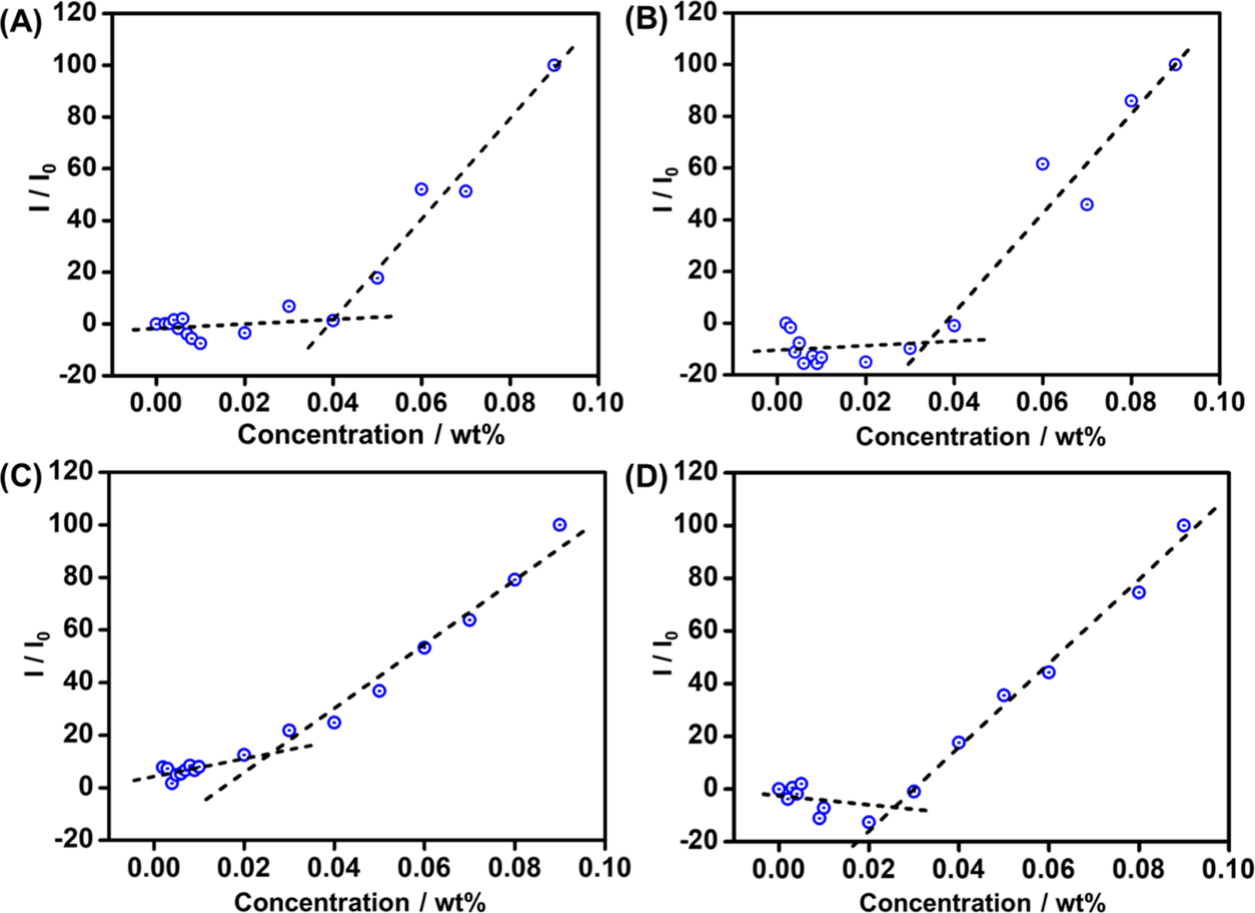
CAC study using ThT peak intensity (λ_max_ = 487 nm) of lipopeptides (A) **P1**, (B) **P1D**, (C) **P2**, (D) **P2D**.

To determine the CAC values of these lipopeptides, fluorescence probe assays using Thioflavin T (ThT) were performed, this dye being sensitive to the formation of β-sheet structures.^44−46^ Emission spectra of the four lipopeptides were collected at different concentrations after excitation at 440 nm. At higher concentrations, an emission peak at 487 nm is visible. By plotting the fluorescence intensity (*I*/*I*_0_) at 487 nm as a function of the concentration, the concentration at the breakpoint (corresponding to the CAC) was found to be 0.040 ± 0.003 wt % for **P1** (Figure 3A), 0.034 ± 0.005 wt %, for **P1D** (Figure 3B), 0.026 ± 0.004 wt % for **P2** (Figure 3C), and 0.026 ± 0.004 wt % for **P2D** (Figure 3D). The lower CAC values for **P2** and **P2D** are due to the higher hydrophobicity of tryptophan compared to tyrosine. These CAC values of the lipopeptides determined by the ThT assay at pH 8 are similar to those obtained at pH 4.6 from surface tension and electrical conductivity measurements,^54^ although they are significantly higher than those obtained from ANS [8-anilinonaphthalene-1-sulfonic acid] fluorescence.^43^ However, since aggregation at the two different pH values gives rise to distinct nanostructures, this may be coincidental.

We examined the kinetics of pH-switching (of secondary structure and nanostructure), by performing a ThT fluorescence kinetic study of the lipopeptides while varying the pH. For this, 0.1 wt % (above the CAC) lipopeptide solutions in 5 × 10^–3^ ThT were prepared at pH 4.6. After 10 min, the pH was changed to 8 by the addition of a few drops of 1 wt % NaOH solution, with data then collected at this basic pH up to 240 min. At this time, the pH was changed back to pH 4.6 by the addition of a few drops of 1(N) HCl solution. Then, the data was collected for up to 300 min. From the kinetic ThT fluorescence data of four lipopeptides (SI Figure S2), we observed that the fluorescence intensity of all the lipopeptides at pH 4.6 is stable with minimal intensity, as expected in the absence of β-sheet structure. As soon as the pH changes from 4.6 to 8, the fluorescence intensity of all the lipopeptides begins to significantly increase due to the binding of ThT with the newly formed fibrous structure. To investigate the stability of the β-sheet structures structure, the fluorescence was monitored for 230 min at pH 8, with a high fluorescence intensity maintained over the entire period. After 240 min the pH was switched back to pH 4.6, which triggered the rapid (within 5 min) disruption of fiber structures, which was evident from the kinetics plots (SI Figure S2), as the fluorescence intensity returned to a minimum. This data indicates pH switching on a timescale of minutes. The control kinetics experiment with only ThT solution without the lipopeptides was performed and it was observed that by switching the pH from acidic to basic to again acidic there is no influence of pH on the ThT fluorescence intensity (SI Figure S3) and thus the observed changes in the presence of lipopeptides are due to the influence of pH on ThT binding to lipopeptide structures. Unfortunately, the time resolution of these measurements (considering mixing times, etc.) did not enable further detailed kinetic analysis.

**Figure 4:**
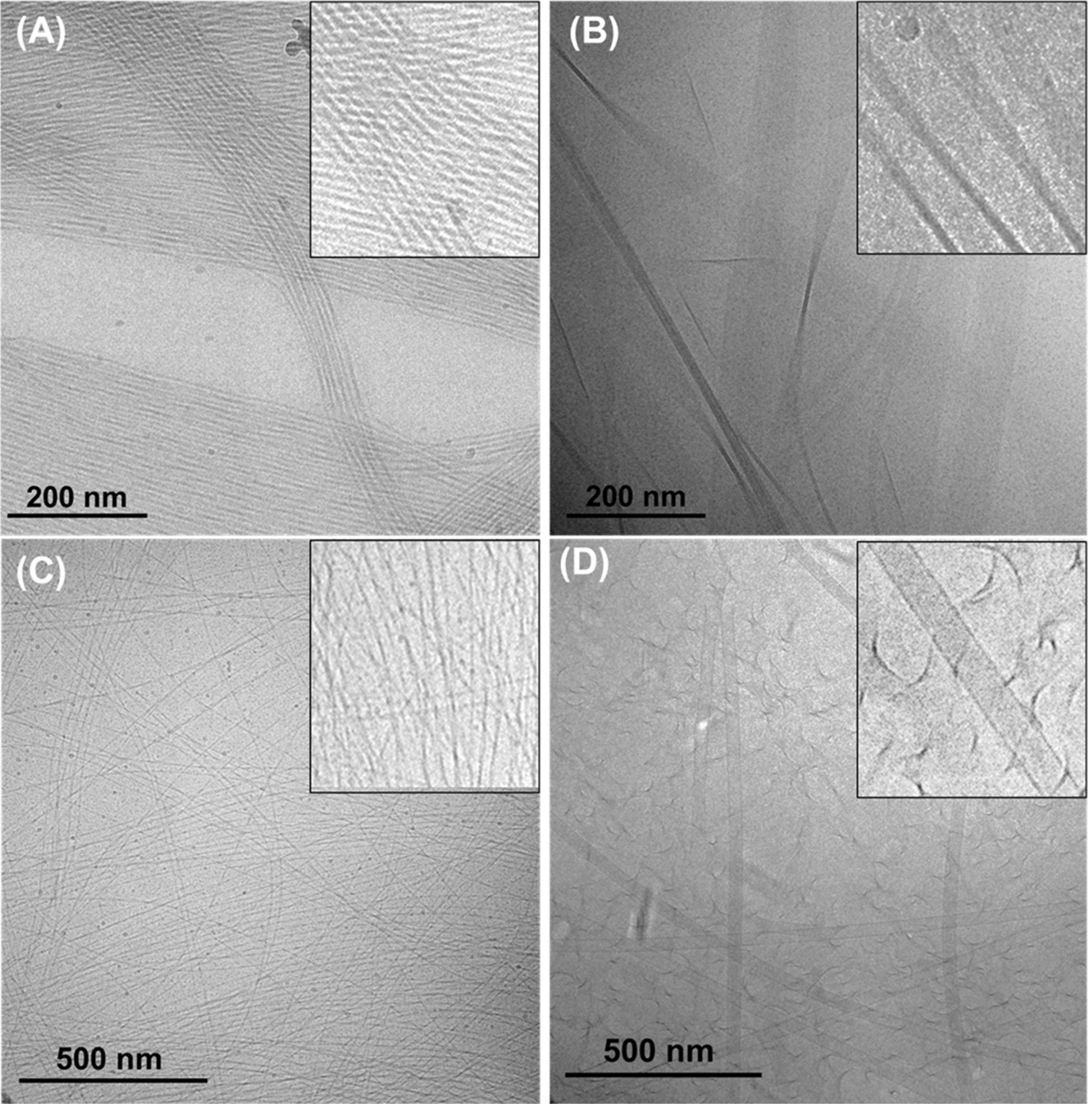
Cryo-TEM images of 1 wt % aqueous solution of lipopeptides at pH 8. (A) **P1**, (B) **P1D**, (C) **P2**, (D) **P2D**.

The self-assembled nanostructure of these lipopeptides at acidic pH was investigated at the lower pH of 4.6 in our recent paper,^43^ and here we report the unexpected observation of distinct self-assembled nanostructure of these lipopeptides at a higher pH. For cryo-TEM imaging at basic pH, 1 wt % aqueous solution of lipopeptides at pH 8 were prepared, this concentration being above the measured CAC values for these lipopeptides. The cryo-TEM images of the samples **P1** and **P2** (Figure 4A,C), reveal a dense network of long intertwined nanofibers with a mean diameter of 11.7 ± 3.3 nm for **P1** and 6.4 ± 2 nm for **P2** respectively. For lipopeptide **P1D**, twisted nanotape structures of variable width (Figure 4B) were observed which notably comprise arrays of individual filaments. For **P2D** lipopeptide, nanotube structures with a mean diameter of 32.8 ± 4.5 nm (Figures 4D and S4) were observed, along with irregular twisted structures. In contrast, the TEM images of all the lipopeptides at pH 4.6 show small spherical micelles with a mean diameter of 4–9 nm (as reported previously,^43^ with additional images in Figure S5).

**Figure 5:**
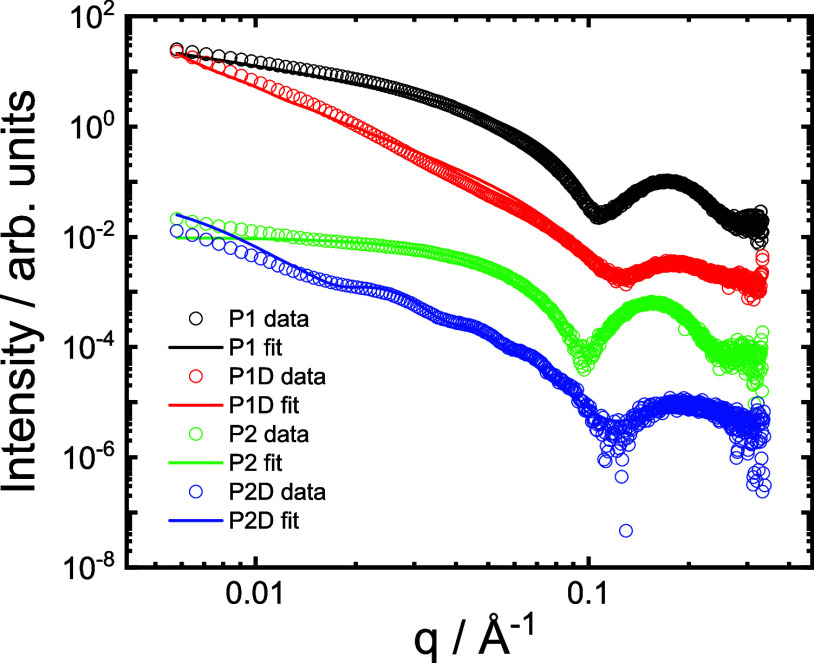
SAXS data for 1 wt % aqueous solution of lipopeptides at pH 8. Open symbols: measured data, Lines: fits as described in the text (fit parameters in SI Table S1). Only every 5th data point is shown for ease of visualization, and data sets are offset vertically for the same reason.

SAXS provides unique in situ information on the internal features of the nanostructures (shape and dimensions).^55^ Synchrotron SAXS data was measured for the four lipopeptides under the same conditions as for the cryo-TEM images. The data are shown in Figure 5, and there are clearly two families of data sets, the shape of the intensity profiles for **P1** and **P2** are similar to each other and are distinct from those for **P1D** and **P2D** which, in some respects, are similar to one another, although there are important differences. The data for **P1** and **P2** could be fitted using a core-shell cylinder form factor, which is consistent with the fibrils observed by cryo-TEM (Figure 4). The corresponding fit parameters are listed in SI Table S1. The core radius *R* is consistent with the length of a C_16_ chain (not fully extended), being *R* = (16.5± 1.0) Å for **P1** and *R* = (13.6 ± 1.0) Å for **P2**. The shell thickness is *s* = 10 or 14.6 Å for **P1** and **P2** respectively, and these values are reasonable for a tripeptide in an extended conformation.

In contrast to the SAXS data for **P1** and **P2**, the intensity curves for **P1D** and **P2D** (Figure 5) have a higher slope at low *q* and broader less intense form factor maxima, at higher wavenumber *q* than the maxima observed for **P1** and **P2**. These features are due to the distinct nanostructures formed by these lipopeptides. Consistent with the cryo-TEM images, the data for **P1D** was fitted using the form factor of a Gaussian bilayer which is used to represent nanotape structures of lipopeptides,^17,56^ since these are built from bilayer structures with an electron density profile represented by three Gaussian functions, one for the central lipid region (with low electron density) and two for the two peptide-covered surfaces (with high electron density). The fit parameters (SI Table S1) indicate a bilayer thickness *t* = (27 ± 4) Å, which indicates highly interdigitated lipopeptides within the bilayers, considering the estimated length of an extended C_16_-tripeptide of approximately this value. For **P2D**, the SAXS data show clear form factor oscillations in the intensity at lower *q*, a characteristic signature of nanotube structures (as revealed by cryo-TEM). Therefore, the data was fitted using a model employed previously for lipopeptide and peptide nanotubes,^17,56^ which includes a core-shell cylinder term for the hollow nanotube plus a Gaussian bilayer term to account for the scattering from the nanotube wall. This represents the data very nicely (Figure 5, SI Table S1 provides a full list of fit parameters) and indicates a nanotube radius *R* = (120 ± 20) Å and a wall thickness *s* = 45 Å. The nanostructure dimension from SAXS may be compared to the cryo-TEM images (Figure 4). Since the measured nanofiber diameters from cryo-TEM images for **P1** and **P2** are significantly larger than the fibril dimensions from SAXS, we conclude that the nanofibers observed by cryo-TEM are bundles of smaller filaments, the size of which is detected by SAXS. Cryo-TEM does not permit accurate measurement of the tape thickness for **P1D**, which is reliably extracted from the SAXS form factor analysis. The nanotube dimensions from cryo-TEM and SAXS for **P2D** are in reasonable agreement. The differences in nanostructure revealed by cryo-TEM images and SAXS data in the case of **P1** and **P2** compared to **P1D** and **P2D**, indicates that the presence of d-lysine in **P1D** and **P2D** alters the molecular packing compared to more flexible l-lysine in the case of **P1** and **P2**, and this change of chirality influences the self-assembled nanostructure. On the other hand, there is a common feature in the sense that changing the pH from acidic (pH 4.6) to basic (pH 8), alters the self-assembled nanostructure of the lipopeptides from micelles to extended structures of various types.

**Figure 6:**
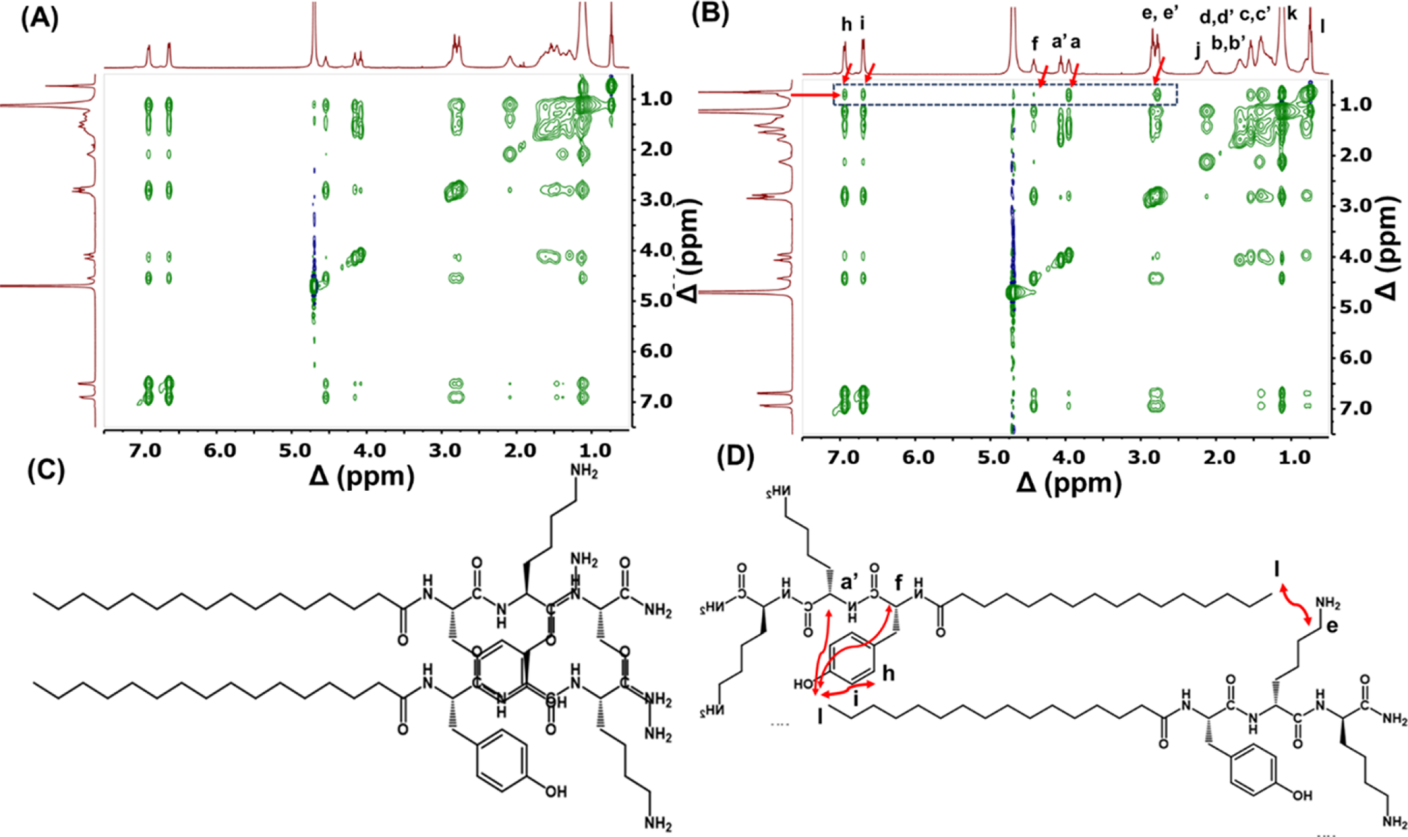
2D NOESY NMR of 1 wt % solution of **P1** (A), **P1D** (B) in D_2_O at pH 8. The possible orientation of lipopeptide strands of **P1** (C), and **P1D** (D).

To understand the arrangement of lipopeptides in the distinct nanostructures observed at pH 8, a 2D NOESY NMR study was carried out by preparing 1 wt % lipopeptides solution in D_2_O. The spectra shown in Figure 6, reveal that extra cross peaks in NOESY were observed for **P1D** compared to **P1**. The extra cross-peaks observed (shown inside the black dotted box with the red arrow in the Figure 6B) are as follows: (1) between the terminal protons of the lipid chain (*l* = 0.73), and aromatic protons (*h* = 6.94 ppm, *i* = 6.69 ppm) of tyrosine, (2) the terminal protons of the lipid chain (*l* = 0.73), and terminal alkyl protons in the side chain of lysine (*e*, *e*′ = 2.77 ppm), (3) the terminal protons of the lipid chain (*l* = 0.73), and α(C–H) of the peptide backbone (*a*, *a*′ = 3.95 ppm, *f* = 4.42 ppm). These cross peaks are absent in the case of **P1** (Figure 6A). This result signifies strong correlations among these protons in space between two strands of **P1D** lipopeptides compared to **P1**, which indicates head-to-tail antiparallel β-sheet stacking of lipopeptide **P1D** (Figure 6D), and parallel β-sheet stacking of lipopeptide **P1** (Figure 6C).

**Figure 7:**
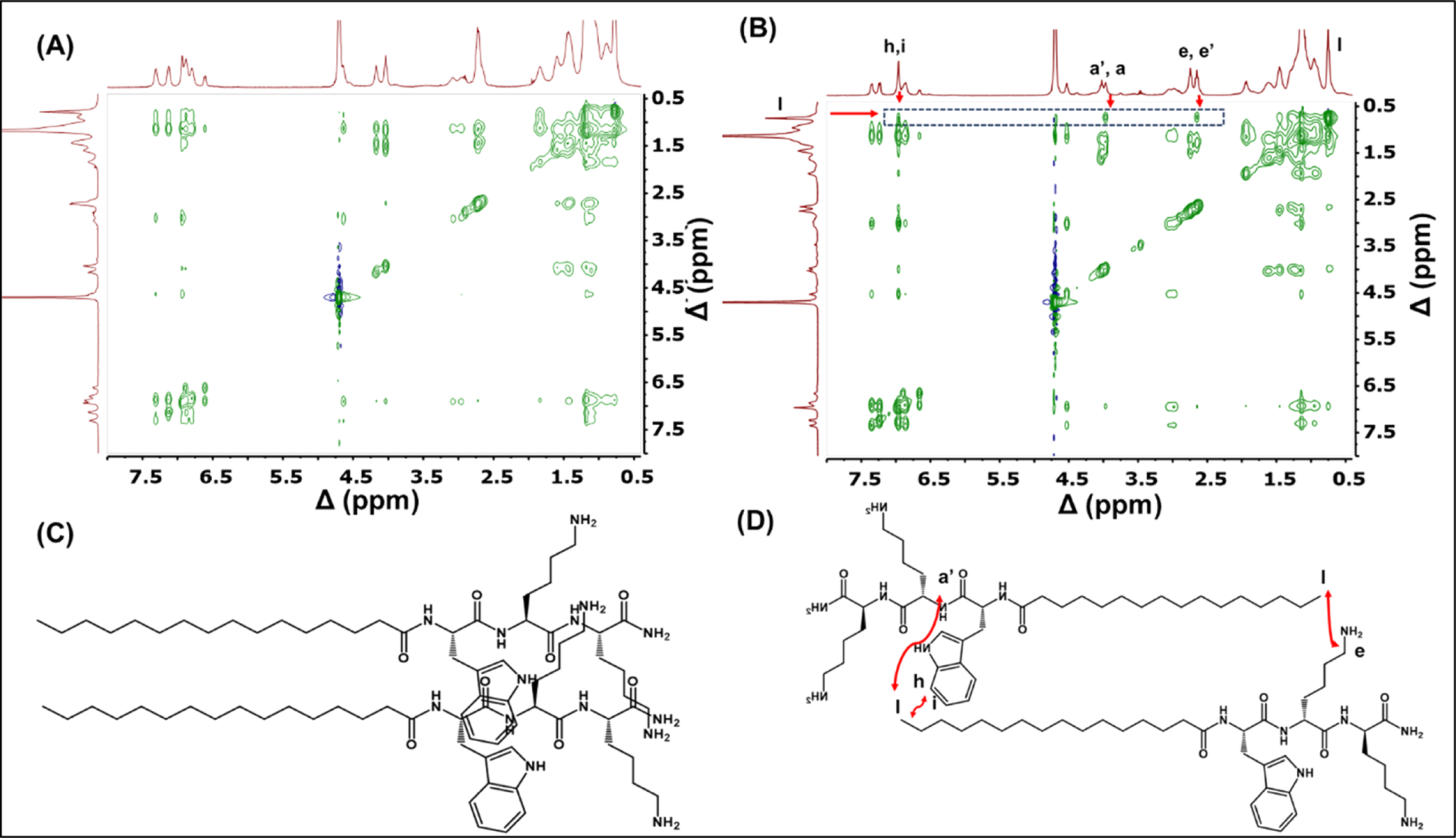
2D NOESY NMR of 1 wt % solution of **P2** (A), **P2D** (B) in D_2_O at pH 8. The possible orientation of the lipopeptide strands of **P2** (C), and **P2D** (D).

**Figure 8:**
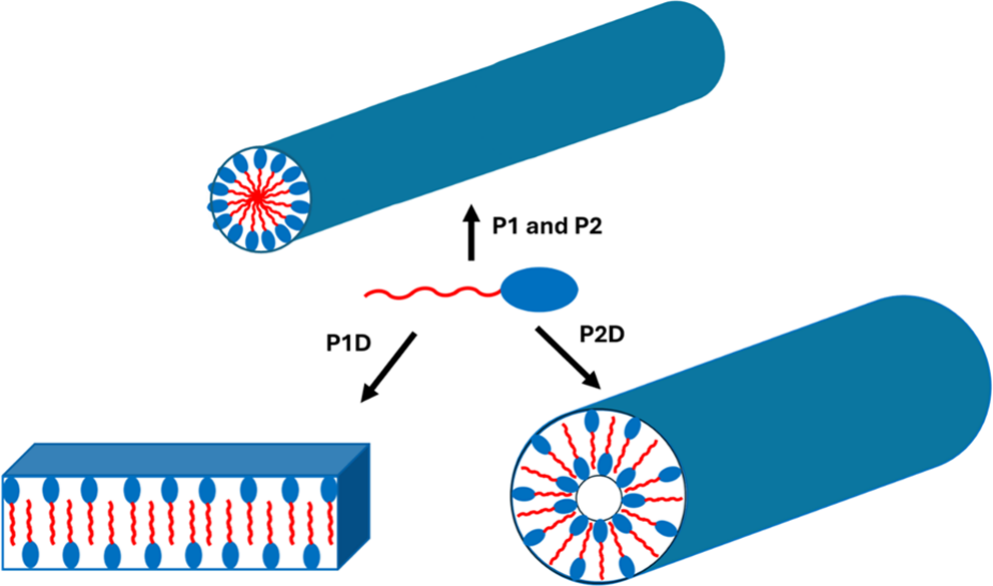
Proposed molecular packing of the lipopeptides **P1**, **P2**, **P1D**, and **P2D** at pH 8. In the cartoon, the blue ellipse indicates hydrophilic peptide, and the red chain indicates hydrophobic lipid.

The same phenomenon was also observed in the 2-D NOESY NMR spectra comparing **P2** and **P2D**. From Figure 7B, the extra cross peaks observed are as follows: (1) between the terminal protons of the lipid chain (*l* = 0.73), and aromatic protons (6.96 ppm) of tryptophan, (2) the terminal protons of the lipid chain (*l* = 0.73), and terminal alkyl protons in the side chain of lysine (*e*, *e*′ = 2.65 ppm), (3) the terminal protons of the lipid chain (*l* = 0.73), and α(C–H) of the peptide backbone (*a*, *a*′ = 3.97 ppm). These cross peaks are absent for **P2** (Figure 7A) signifying strong correlations among these protons in space between two strands of **P2D** lipopeptides compared to **P2**, which indicates head-to-tail antiparallel β-sheet stacking of lipopeptides **P2D** (Figure 7D), and parallel β-sheet stacking of lipopeptides **P2 (**Figure 7C**)**. The proposed arrangement of **P1** and **P2** agrees with the SAXS data which indicates individual fibrils comprise a lipid core surrounded by a tripeptide corona. Similarly, the arrangement of **P1D** and **P2D** deduced from NOESY is consistent with a highly interdigitated antiparallel packing deduced from SAXS form factor analysis for the nanotape/nanotube structures. The self-assembly of the homochiral lipopeptides **P1** and **P2** at pH 8 results from the parallel β-sheet stacking of lipopeptides, which results in the formation of nanofibers, as represented in the schematic in Figure 8. In contrast, the self-assembly of heterochiral lipopeptides **P1D** and **P2D** at pH 8 is due to antiparallel β-sheet stacking of lipopeptides, which results in the formation of nanotapes and nanotubes respectively, as illustrated in Figure 8.

**Figure 9:**
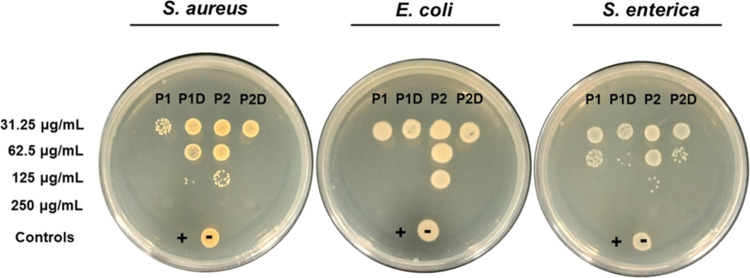
Determination of MBC of lipopeptides **P1, P1D, P2**, and **P2D**. 10 μL aliquots from broth microdilution plates were spot-inoculated onto LB agar plates. The MBC refers to the lowest concentration of an antibacterial agent required to completely kill bacteria shown by the lack of colony formation. No visible bacterial colony-forming units were observed at 250 μg/mL for all strains tested. Positive control (+) 0.97 μg mL^–1^ ciprofloxacin; negative control (−) 1X PBS.

Lysine-rich peptides and lipopeptides are promising biomaterials with potential antimicrobial activity. The antibacterial activity of the lipopeptides was investigated by measuring the Minimum Bactericidal Concentration (MBC) of lipopeptides **P1**, **P1D**, **P2**, and **P2D** against *E. coli*, *S. aureus*, and *S. enterica*. From the MBC assay, it was observed that all the lipopeptides showed significant inhibition towards bacterial growth, and the minimum bacterial concentration was in the range 62.5–250 μg/mL (Figure 9 and SI Table S2). Trends that are apparent include a lower activity for **P2** for all species and a generally lower activity against *S. enterica* for all lipopeptides. The MBC values may be compared with our previously reported MIC (minimum inhibitory concentration) values (reproduced in Table 1 for convenience).^43^ A notable trend comparing the activity of lipopeptides to each other is for the MIC values for **P1** and **P1D** to be lower compared to **P2** and **P2D** except against *S. aureus*. Again, there seems to be a general tendency for lower activity (higher MIC values) of a given lipopeptide for *S. enterica*.

**Table 1:** Antimicrobial, Cytotoxic, and Selectivity Index Activities of Lipopeptides^a^

	MIC (μg/mL)				SI		
lipopeptide	*E. coli*	*S. enterica*	*S. aureus*	HC_50_ (μg/mL)	*E. coli*	*S. enterica*	*S. aureus*
**P1**	15.63–31.25	31.25–62.5	31.25–62.5	80.90 ± 0.89	2.58–5.17	1.29–2.58	1.29–2.58
**P1D**	15.63–31.25	31.25	31.25	78.16 ± 1.10	2.5–5	2.5	2.5
**P2**	15.63–62.5	62.5–250	31.25–62.5	38.81 ± 1.13	0.62–2.33	0.15–0.62	0.62–1.24
**P2D**	31.25–62.5	62.5	31.25–62.5	55.46 ± 0.81	0.88–1.77	0.88	0.88–1.77

a MIC values were reported previously.^43^

To further examine bioactivity, the cytotoxicity of the lipopeptides was investigated by performing hemocompatibility assays on hRBCs. For practical, and eventual clinical, application as antimicrobial agents, compounds must lie within an acceptable hemocompatibility range and exhibit good selectivity. For the hemolysis assay, hRBCs were incubated with increasing concentrations (15.62–1000 μg mL^–1^) of **P1**, **P1D**, **P2**, and **P2D** for 1 h. Dose-dependent erythrocyte lysis was observed (SI Figure S6). **P1** and **P1D** have similar hemolytic properties, reaching 50% of cell lysis in comparable ranges: 80.90 ± 0.89 and 78.16 ± 1.10 μg mL^–1^, respectively (Table 1). Conversely, **P2** showed moderate hemolysis with HC_50_ = 38.81 ± 1.13 μg mL^–1^ and HC_50_ = 55.46 ± 0.81 μg mL^–1^ for **P2D**. Here 1% Triton X-100 was considered as a positive control, which promotes rapid cell lysis because of its surfactant nature (SI Figure S6). The analysis of the selectivity index (SI) provides valuable insight into the potential use and safety of a therapeutic candidate for successful translation as an antimicrobial. Higher values are usually associated with greater therapeutic benefits and reduced side effects making them more attractive for application in clinical settings. In this context, the data in Table 1 show that **P1** and **P1D** have SIs between 2.5 to 5.17 for *E. coli*, and 1.29 to 2.58 for *S. aureus*. In contrast, **P2** and **P2D** showed significantly lower SIs ranging between 0.15 to 1.77 for all tested bacterial strains (Table 1). Selectivity profiling using hRBCs indicates that all lipopeptides have a higher preference for prokaryotic cells with minimal off-target activity at the bactericidal concentration.

While d-isomers of lysine appear to have no effect on reducing hemolysis when paired with a tyrosine residue (comparing hemolysis for **P1** and **P1D**, Table 1), their combination with tryptophan in **P2D** showed a slight decrease in toxicity compared to **P2**. Previous reports have highlighted the impact of chirality on the cytotoxicity and antimicrobial properties of peptides.^57,58^ The distinct combination of chirality of d-lysine along with the non-polar aromatic ring in l-tryptophan, leads to unique three-dimensional conformations which can influence the interaction of the peptides with eukaryotes compared to prokaryotes. It is widely recognized that these types of organism exhibit distinctive profiles in membrane composition; for instance, eukaryote membranes predominantly contain cholesterol as sterol, whereas prokaryote membranes typically contain ergosterol.^59^ In addition, it has been demonstrated that tryptophan residues can bind, through hydrogen bonds, with lipid and cholesterol chains present in the cell membrane, which can result in changes in fluidity.^60^ Although erythrocytes lack cholesterol in their membrane composition, tyrosine paired with d-lysine in **P1D** seems to enable selective interaction with prokaryotes, resulting in an expanded therapeutic window, effectively balancing antimicrobial activity and cytotoxicity. Several d-amino acid- containing peptides were observed to be highly selective at targeting bacteria.^61,62^ Zhao et al. have demonstrated that a short cationic peptide d-lysine-MPI not only displayed more potent antimicrobial activity than its parent Polybia-MPI (extracted from social wasp Polybia paulista) but also reduced toxicity to erythrocytes.^63^ While some peptides containing d-amino acids seem to be attractive candidates due to higher selectivity and reduced in vivo proteolysis of d-amino acid peptides, the fine balance between targeting bacteria over mammalian cells must be considered in pre-clinical discovery projects of novel lipopeptide-based antibiotics. Thus, future studies focusing on activity-structure optimizations using these templates can lead to the development of more selective analogues with a higher capacity to differentiate human cells and bacteria, reducing further the probability of inducing unwanted side effects. Earlier studies analyzing the selectivity of lytic lipopeptides have pointed out significant differences in membrane-lipopeptide interactions with bacteria and human cells due to contrasting phospholipid composition.^64−69^ The higher electrostatic/hydrophobic recognition of microbial membranes by lipopeptides has been suggested as the basis of the formation of pores or other membrane damage associated with increased permeability and functional consequences, including bacterial death. To clarify the action underlying the killing effect of lipopeptides, we explored in vitro activity on membrane permeation using clinically relevant bacteria.

**Figure 10:**
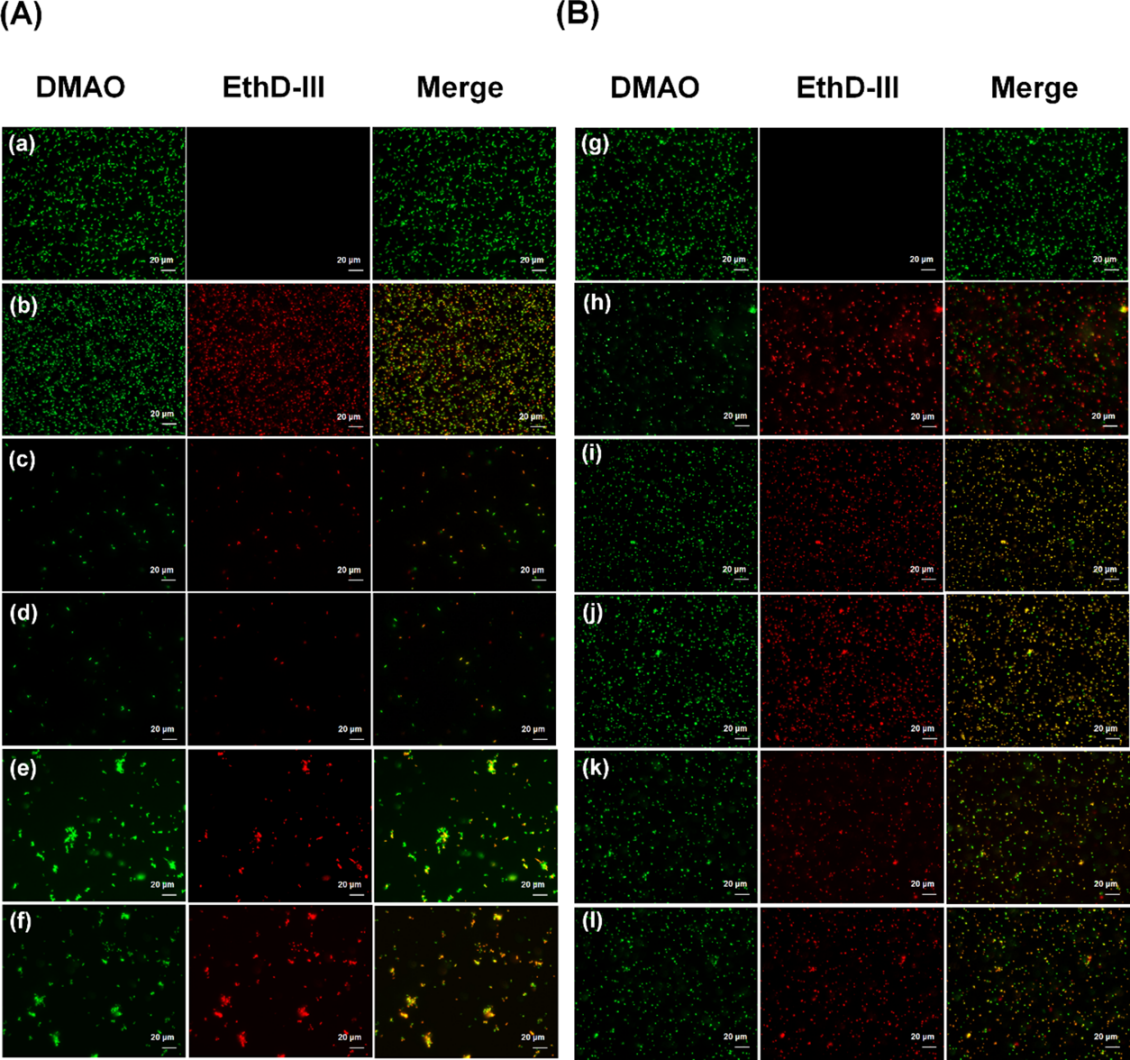
Membrane permeabilization of *E. coli* (A) and *S. aureus* (B) was observed through epifluorescence microscopy. Bacterial cells were incubated with two-fold MIC of lipopeptides for 1 h at 37 °C followed by live/dead staining (Promokine). With appropriate mixtures of both dyes, live bacterial cells with intact membranes stain green (DMAO) whilst non-viable bacterial cells with disrupted membranes stain red (EthD-III). Merge (orange) represents the superposition of both dyes (combined viable and non-viable cells). (a, g) PBS negative controls; (b, h) Triton X-100 positive controls; (c, i) bacterial cells incubated with **P1**; (d, j) bacterial cells incubated with **P1D**; (e, k) bacterial cells incubated with **P2**; (f, l) bacterial cells incubated with **P2D**.

To complement our previous cell viability studies (MTT assays),^43^ differential epifluorescence was imaged using a Promokine Bacterial Live/Dead Stain kit. The results shown in Figure 10 suggest that lipopeptides disrupt membrane integrity and compromise the viability of both Gram-negative bacteria and Gram-positive strains tested. Peptide-treated cells showed significant red fluorescence indicating binding of the EtD-III dye to bacterial DNA of both *E. coli* and *S. aureus* (Figure 10) strongly suggesting rupture of bacterial cellular membranes. The formation of cell aggregates (clusters) was observed for **P2** and **P2D** for *E. coli* when compared to **P1** and **P1D**. Conversely, in control experiments, no effect on viability (i.e., all cells fluoresced green) was observed when bacteria were incubated solely with PBS.

## Conclusions

In conclusion, to understand the influence of chirality and pH on the self-assembly process, a small library of homochiral and heterochiral lipidated tripeptides was designed. Consistent with our previous report,^43^ we find that under acidic pH conditions, all the lipopeptides self-assemble into micelles. In contrast at basic pH the homochiral lipopeptides self-assemble into nanofibers, whereas the heterochiral lipopeptide containing tyrosine self-assembles into nanotapes and the heterochiral lipopeptide containing tryptophan self-assembles into nanotubes, as shown by a combination of cryogenic-transmission electron microscopy (cryo-TEM) and small-angle X-ray scattering (SAXS). Concomitant changes in molecular conformation were probed by spectroscopic methods which show that the extended structures contain intermolecular hydrogen-bonded β-sheet structures. The formation of distinct extended nanostructures for the l-lysine lipopeptides compared to the d-lysine lipopeptides was explained based on contrasting β-sheet packing (parallel strands for fibrils, antiparallel strands for nanotapes and nanotubes) based on 2D NMR. Thus, chirality and/or pH in lipopeptides can be used to tune the self-assembly behavior. These lipopeptides also show promising bioactivity, with good cytocompatibility in hemolytic assays and antimicrobial activity against both Gram-negative and Gram-positive bacteria shown through the determination of MIC and MBC values, and live/dead bacteria staining assays.

## Supporting Information Available

FTIR spectra, ThT fluorescence pH switch kinetic data, cryo-TEM images at pH 4 and additional cryo-TEM image for P2D, hemolytic assay data, tables of SAXS fit parameters and MBC values (PDF)
