# Supplementary material for: Chirality and pH Influence the Self-Assembly of Antimicrobial Lipopeptides with Diverse Nanostructures
Source: ACS Appl Bio Mater. 2024 Jul 23;7(8):5553–65. doi: 10.1021/acsabm.4c00664 (PMC11337160; doi:10.1021/acsabm.4c00664)
Supplement: Supplementary file 1 — mt4c00664_si_001.pdf [file mt4c00664_si_001.pdf]

Supporting Information

Chirality and pH Influence the Self-Assembly of Antimicrobial Lipopeptides with Diverse Nanostructures

Anindyasundar Adak,[1] Valeria Castelletto,[1] Bruno Mendes,[2] Glyn Barrett,[2] Jani Seitsonen,[3] Ian W. Hamley[1,*]

[1] School of Chemistry, Pharmacy and Food Biosciences, University of Reading, Whiteknights, Reading, RG6 6AD, UK

[2] School of Biological Sciences, University of Reading, Whiteknights, Reading, RG6 6AH, UK

[3] Nanomicroscopy Center, Aalto University, Puumiehenkuja 2, FIN-02150 Espoo, Finland

* Author for correspondence. I.W.Hamley@reading.ac.uk

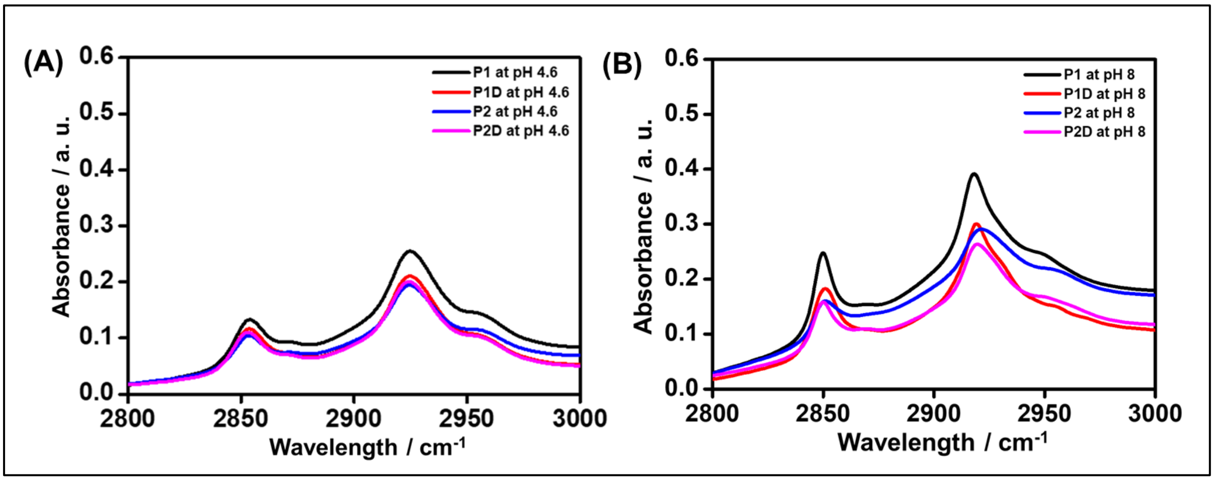

Figure S1. FTIR spectra (methyl/methylene band region) of 1 wt% aqueous solution of lipopeptides (A) pH 4.6, (B) pH 8.

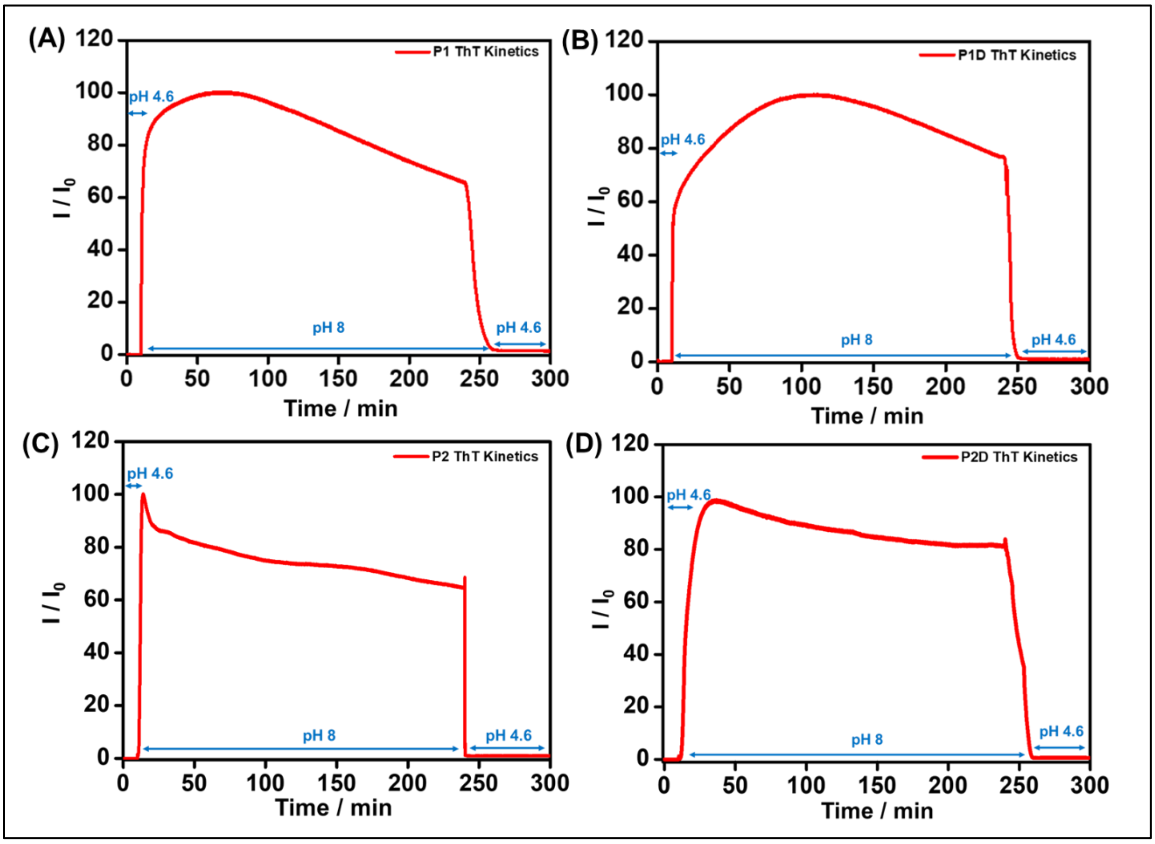

Figure S2. ThT kinetics study (using ThT peak intensity at λ_{max} = 487 nm) of lipopeptides (A) **P1**, (B) **P1D**, (C) **P2**, (D) **P2D**.

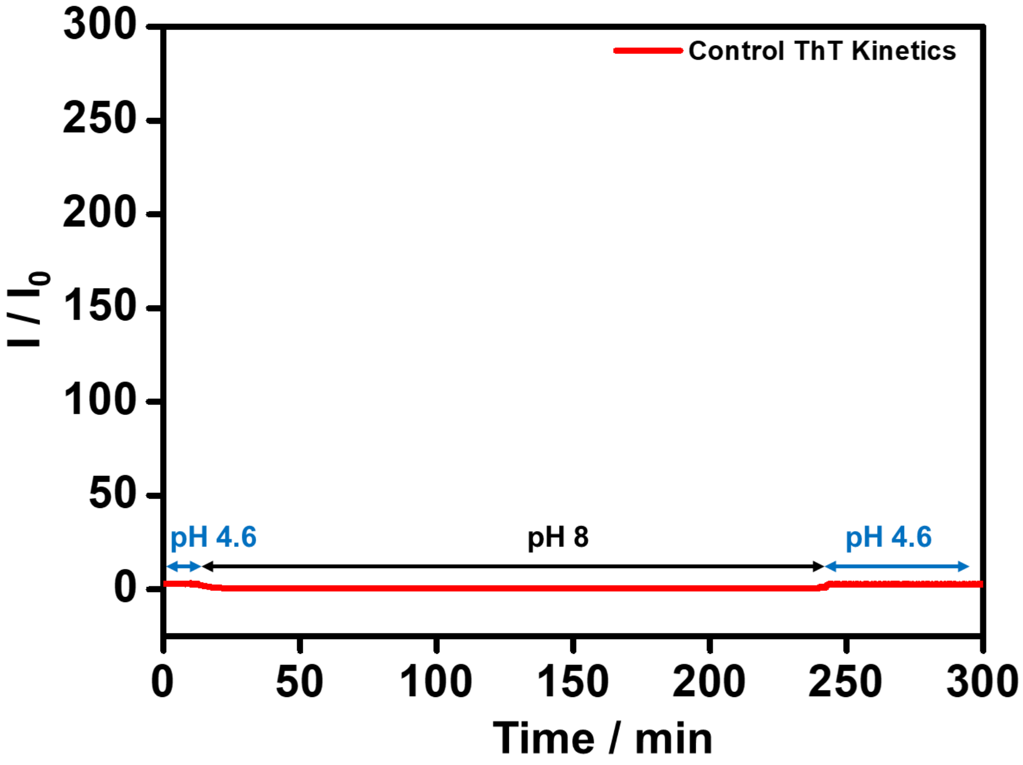

Figure S3. Control kinetics study with varying pH: (using ThT peak intensity at λ_{max} = 487 nm) of ThT without lipopeptides.

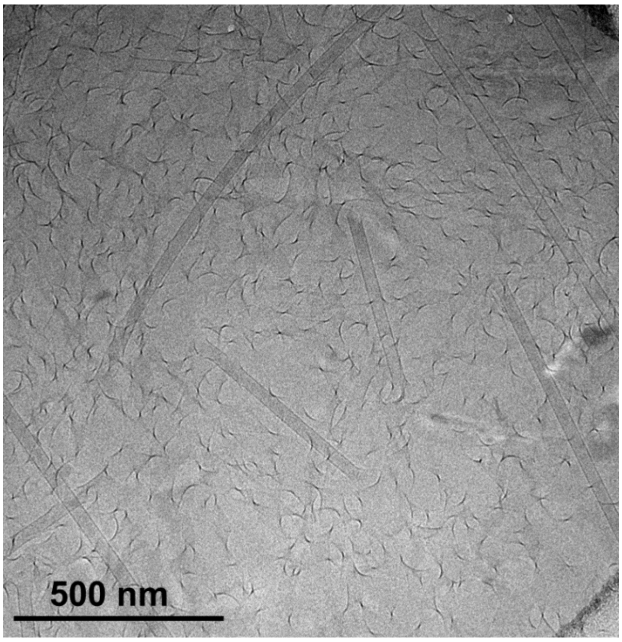

Figure S4. Additional Cryo-TEM image of 1 wt% aqueous solution of lipopeptide **P2D** at pH 8.

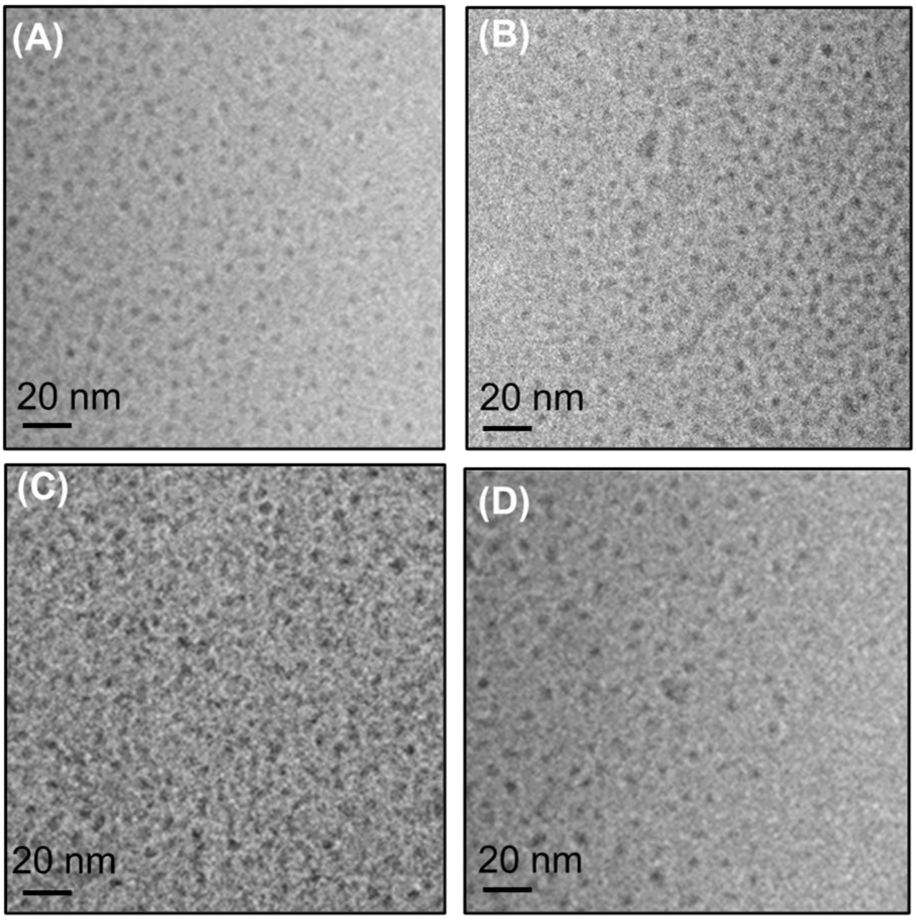

Figure S5. Cryo-TEM images of 1 wt% aqueous solution of lipopeptides at pH 4.6 (A) **P1**, (B) **P1D**, (C) **P2**, (D) **P2D**.

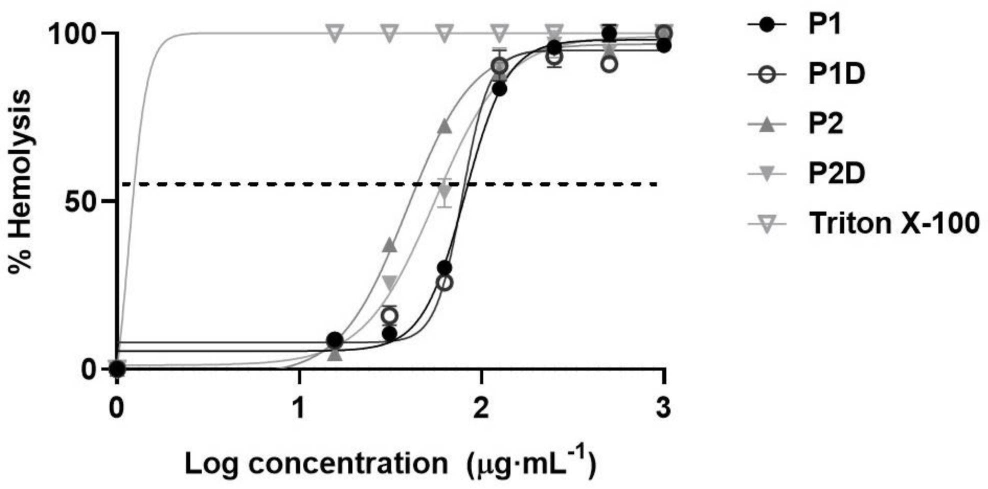

Figure S6. Hemolysis profile showing induced dose-dependent lipopeptides. The hRBC suspension was incubated with increased concentrations of **P1**, **P1D**, **P2**, and **P2D** (15.62 -1000 μgmL^{-1}) for 1 h at 37 ^{o}C, and 1 % Triton X-100 was used as a positive control. The optical density of the supernatant was measured at 414 nm. Values are plotted as mean ± standard error (n = 3).

Table S1. Parameters extracted from the fitting of the SAXS data for 1 wt% solutions at pH 8.[a]

	**P1**<br>*Long cylindrical shell*	**P1D**<br>*Bilayer*	**P2**<br>*Long cylindrical shell*	**P2D**<br>*Bilayer+ Long cylindrical shell (for nanotube)*
w_1				2.42
$t \pm \Delta t$ [Å]		27.0 ± 4.0		21.0 ± 0.5
η_{out}		3.64×10^{-7}		2.00×10^{-7}
σ_{out} [Å]		3.7		8.0
η_{in}		4.93×10^{-8}		-1.82×10^{-7}
σ_{in} [Å]		5.5		5.5
D [Å]		1200		800
w_2				0.36
R [Å]	16.5 ± 1.0		13.6 ± 1.0	120 ± 20
s [Å]	10.0		14.6	45
η_{core}	1.00×10^{-7}		2.43×10^{-6}	5.00×10^{-8}
η_{shell}	1.00×10^{-6}		-3.58×10^{-6}	-1.52×10^{-7}
η_{solv} [b]	1.00×10^{-9}		1.00×10^{-8}	5.00×10^{-8}
L	2000		89.8	1000
C	0.002	0.0008	0.002	0.00242
B		1.00×10^{-6}		
n		3.2		

[a] Data fitted using the software SASfit.[1-2]

[b] Fixed Parameter

Key: Gaussian bilayer: layer thickness t (Gaussian polydispersity Δt), scattering contrast of outer layers η_{out}, and inner layer η_{in}, Gaussian widths σ_{in} and σ_{out} of inner and outer layers respectively, D diameter (width) of layer system (when $D >> t$ as here, it acts as a scaling parameter for the form factor). **Long cylindrical shell:** R core radius (Gaussian polydispersity Δt), s shell thickness, scattering contrasts of core η_{core}, shell η_{shell} and solvent η_{solv}, L length. **Background:** constant background, C or including sloping background $C + BI^{-n}$. Weightings for two-component form factors, w_1, w_2.

Table S2. Minimum bactericidal concentration (MBC) values of lipopeptides **P1, P1D, P2,** and **P2D**

	**MBC [µg/ml]**		
**Lipo peptide**	***S. aureus***	***E. coli***	***S. enterica***
**P1**	62.5	62.5	125
**P1D**	250	62.5	125
**P2**	250	250	250
**P2D**	62.5	62.5	125

References

(1) Bressler, I.; Kohlbrecher, J.; Thünemann, A. F., SASfit: a tool for small-angle scattering data analysis using a library of analytical expressions. *J. Appl. Cryst.* **2015,** *48*, 1587-1598.

(2) Kohlbrecher, J.; Bressler, I., Updates in SASfit for fitting analytical expressions and numerical models to small-angle scattering patterns. *J. Appl. Cryst.* **2022,** *55*, 1677-1688.
